# Evaluation of cerebral circulation during retrograde perfusion by laser speckle flowgraphy

**DOI:** 10.1007/s11748-016-0727-z

**Published:** 2016-11-29

**Authors:** Fumiaki Kimura, Hirotsugu Kanda, Yuki Toyama, Takayuki Kunisawa, Taiji Nagaoka, Akitoshi Yoshida, Hiroto Kitahara, Hiroyuki Kamiya

**Affiliations:** 1 Department of Cardiac Surgery, Asahikawa Medical University, Midorigaoka Higashi 2-1-1-1, Asahikawa, 078-8510 Japan; 2 Department of Anesthesiology and Critical Care Medicine, Asahikawa Medical University, Midorigaoka Higashi 2-1-1-1, Asahikawa, 078-8510 Japan; 3 Department of Ophthalmology, Asahikawa Medical University, Midorigaoka Higashi 2-1-1-1, Asahikawa, 078-8510 Japan

**Keywords:** Cerebral circulation, Laser speckle flowgraphy, Retrograde cerebral perfusion

## Abstract

Laser speckle flowgraphy (LSFG) is an ophthalmologic equipment that qualitatively detects the blood flow of the optic nerve head, which is known to be related with cerebral microcirculation. LSFG can also measure the mean blur rate, which quantitatively calculates the blood flow. We aimed to assess the utility of LSFG in the evaluation of cerebral perfusion during aortic surgery under hypothermic circulatory arrest with retrograde and antegrade cerebral perfusion. Two patients underwent total arch replacement for aneurysm. The blood flow of the optic nerve head was monitored with LSFG and the mean blur rate value was measured during the surgery. The LSFG could detect the blood flow quantitatively in the optic nerve head during both retrograde and antegrade cerebral perfusion; and the value was correlated with rSO2 value.

## Introduction

Laser speckle flowgraphy (LSFG) is a novel technology used in evaluating the blood flow of the optic nerve head (ONH) [1]. Because the ophthalmic artery is the first branch of the internal carotid artery distal to the cavernous sinus, the blood flow of the ONH reflects cerebral microcirculation [2]. The specification and efficacy of LSFG have been described in ophthalmology [3]. LSFG allows qualitative and quantitative estimation of blood flow in the ONH through the laser speckle phenomenon. Compared with other laser-based techniques (i.e., laser Doppler velocimetry and flowmetry), LSFG measurement covers a larger field and enables two-dimensional observation of the overall hemodynamic condition of the tissue. The laser speckle phenomenon is an interference event occurring when coherent light sources are scattered by the diffusing surface. The speckle pattern, which appears under the illumination of laser irradiation, can be described statistically. In accordance with the movement of red blood cells in the tissue, the structure of the speckle pattern varies rapidly depending on blood flow velocity. In the present study, the mean blur rate (MBR) was obtained by LSFG-NAVI (Softcare, Fukuoka, Japan) and used as an indicator of blood flow speed. We have focused on the potential of LSFG as a new method of neuro-monitoring during cardiac surgery. In this study, we performed two aortic operations with retrograde and antegrade cerebral perfusion (RCP and ACP, respectively) under hypothermic circulatory arrest (HCA) with LSFG measurement of cerebral perfusion.

## Cases

### Surgical procedures

Patient 1, 68-year-old woman, and patient 2, 80 year-old man underwent total arch replacement because of aneurysm of the aortic arch. Pre-operative computed tomography detected no atherosclerotic lesions in the internal carotid artery in both patients. After median sternotomy, cardiopulmonary bypass (CPB) was established with arterial cannulation into the ascending aorta and venous cannulation into the superior and inferior vena cava. After cooling the core temperature to 26 °C, circulatory arrest was induced and RCP was simultaneously started through the venous cannula into the superior vena cava, keeping a venous pressure of 20 mmHg. Cardioplegic solution was retrogradely and simultaneously administered through the coronary sinus during this time point. After inspection of the inner lumen of the aortic arch, the RCP was stopped, three cannulas were inserted into the arch vessels, and ACP was started. Arterial blood pressure was measured in bi-lateral radial artery and cannula tip in left common carotid artery, and maintained up to 50 mmHg. Blood flow was perfused at 300 ml/min in brachiocephalic artery, 150 ml/min in left common carotid and left subclavian artery, respectively. For the reconstruction of the aortic arch, a four-branched prosthesis was used. After distal anastomosis with the descending aorta was performed, perfusion of the lower body was restarted via a branch of the prosthesis. Thereafter, proximal anastomosis with the ascending aorta was performed and the patients were re-warmed. Three arch vessels were anastomosed with branches of the prosthesis. After applying the anastomosis, the patients were immediately weaned from the CPB.

### LSFG measurement

**Fig. 1:**
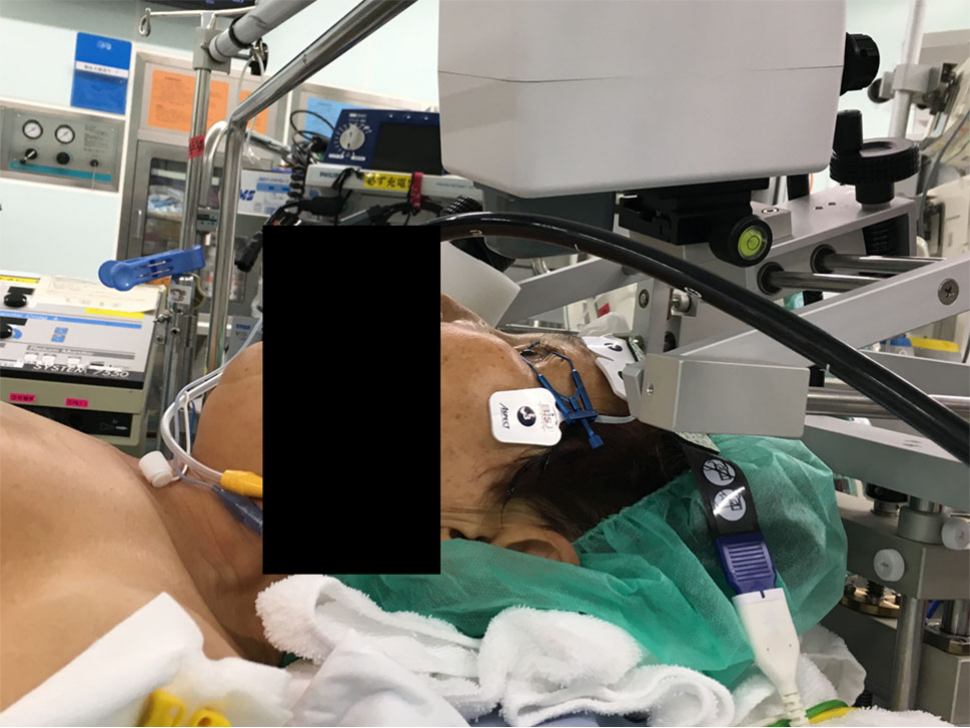
The laser speckle flowgraphy equipment was set on the patient’s* left eye* during surgery

**Fig. 2:**
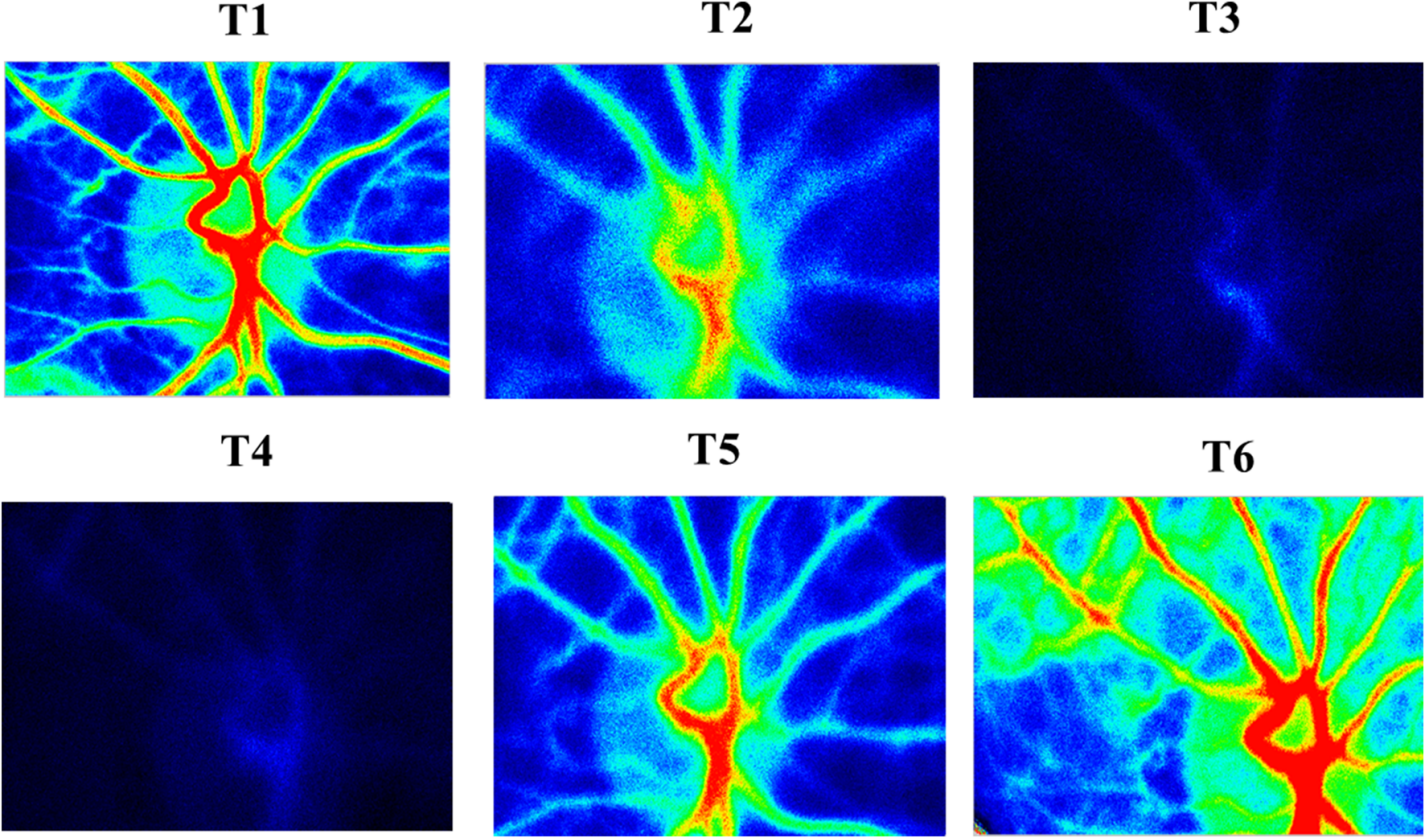
Laser speckle flowgraphy showing retinal blood flow map in patient 1. *Red field*, area with higher blood flow

**Fig. 3:**
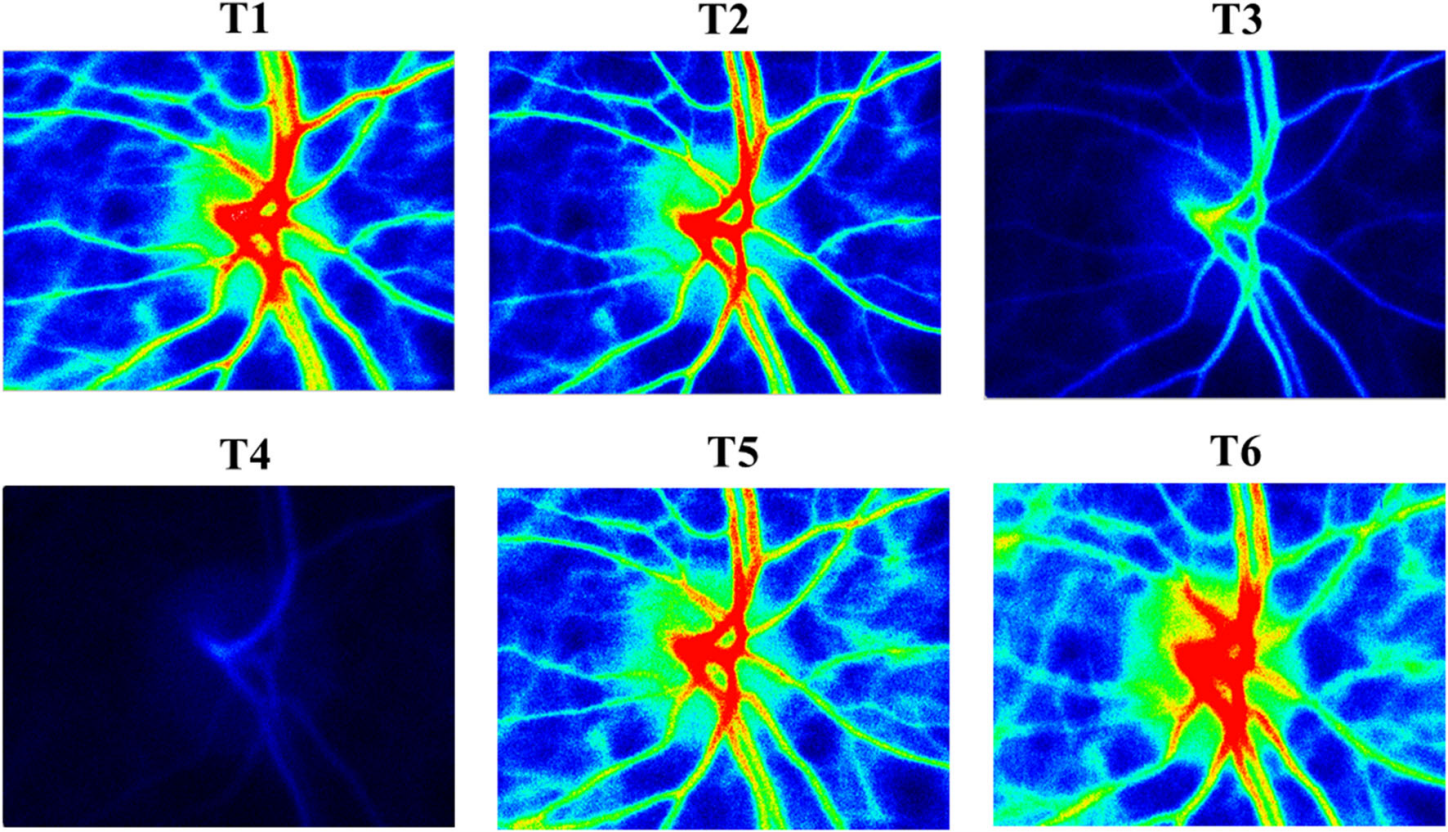
Laser speckle flowgraphy in patient 2. *Red field*, area with higher blood flow

**Fig. 4:**
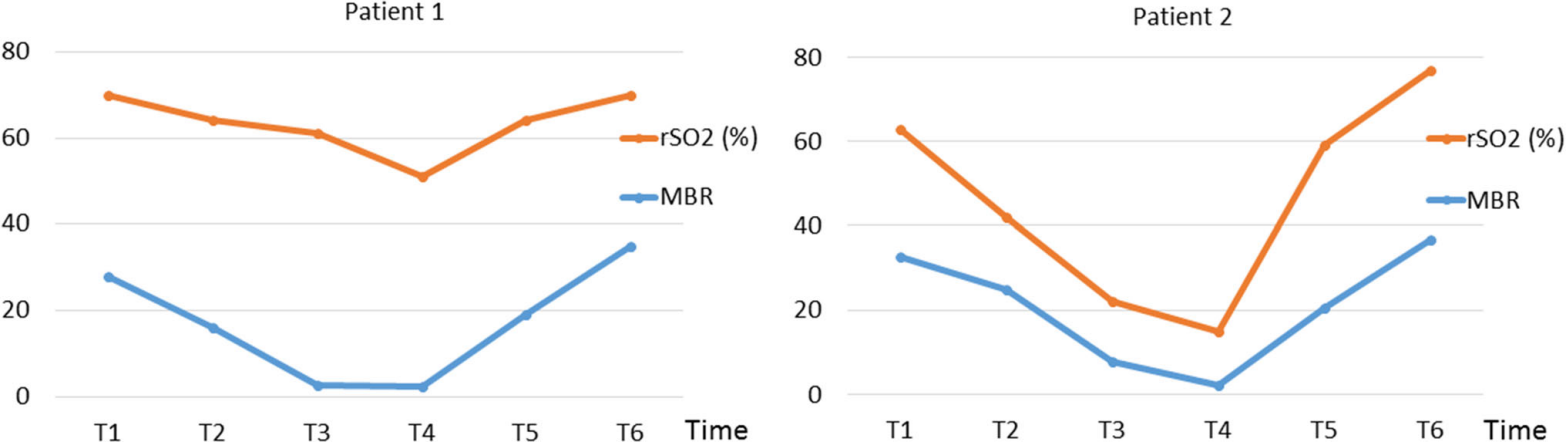
The time course of the mean blur rate (MBR) value and regional cerebral oxygen saturation value (rSO_2_) during surgery in two patients

After induction of general anesthesia, the LSFG equipment was set on the left eye (Fig. 1). During the measurement time, an eyelid opener was attached and mydriasis was induced by the administration of eye drops containing 0.5% tropicamide and 0.5% phenylephrine hydrochloride (Mydrin-P ophthalmic solution; Santen Pharmaceutical Co., Ltd., Osaka, Japan). LSFG measurement was performed at six time points through the operation: after inducing general anesthesia (T1), after establishment of CPB with heart beating (T2), 3 min after initiation of RCP with HCA (T3), at the time of HCA without any adjunct cerebral perfusion (T4), 3 min after initiation of ACP with HCA (T5), and after weaning from CPB (T6). It took only a few seconds to measure in each time. T4 time was approximately 10–20 s in both patients. The regional cerebral oxygen saturation (rSO_2_) was continuously measured during the operation with near-infrared spectroscopy (NIRS) (INVOS 5100C; Covidien, Minneapolis, MN). The results of the qualitative analysis of LSFG measurement in patients 1 and 2 are shown in Figs. 2 and 3. The red field indicates the area with higher blood flow. The photographic data were analyzed with LSFG-NAVI, and the results were presented as MBR value. The MBR values in patient 1 were 27.9, 16.1, 2.5, 2.3, 19.2, and 34.8, and those in patient 2 were 32.7, 25, 7.9, 2.3, 20.5, and 36.6 at T1, T2, T3, T4, T5, and T6, respectively. Figure 4 shows the time course of the MBR and rSO_2_ values in two patients. The clinical courses of both participating patients were uneventful without any complication. The institutional review board approved this study, and informed consent was obtained from the participating patients.

## Discussion

The crucial finding of this pilot study was that, for the first time, cerebral microcirculation during aortic surgery with HCA and RCP, HCA alone, and HCA and ACP could be observed in humans using LSFG.

Several modalities are used to evaluate cerebral perfusion during aortic surgery. However, each modality has its own advantages and disadvantages. The arterial blood pressure is the simplest method in evaluating cerebral perfusion, but it is not useful during HCA or HCA and RCP at all. Currently, rSO_2_ measured with NIRS is one of the widely accepted standard neuro-monitoring methods [4]. However, its technical limitation is that only the surface area of the brain can be evaluated with NIRS. Moreover, NIRS sometimes lacks sensitivity, and perfusion deterioration is normally detected with some delay [5]. Ghazy et al. successfully detected cerebral malperfusion during aortic surgery with ACP using transcranial Doppler ultrasound and optimized inadequate cerebral perfusion [6]. However, because the anatomical position of the median cerebral artery differs in individual patients, quantitative analysis of blood flow is not always possible. Nenekidis et al. [2] reported the measurement of retinal mucosal blood flow during cardiac surgery with a laser Doppler flowmeter. They concluded that the ONH is the optimal window for evaluation of cerebral microcirculation because of its anatomical condition. We agree with their augmentation of the procedure using laser Doppler flowmeter, but it has its own technical limitation. With the laser Doppler flowmeter, only pin-point measurement is possible, which renders the measurement of blood flow inaccurate, especially during aortic surgery because the position of a patient is frequently altered (i.e., head up or head down) for de-airing or optimization of operative field.

We prefer to use both RCP and ACP. One of the reasons is we want to shorten HCA time as short as possible. Soon after the circulatory arrest, RCP can be started easily, compared to ACP. After removing the aorta and detecting the arch vessels, we change to ACP. During this changing time, we shortly use HCA. Another reason is that RCP would be effective for de-air and evacuation of debris from the arch vessels. Many previous studies demonstrated that RCP has positive effect on the prevention of neurological complication in aortic surgery [7]. However, there has been no clear evidence of maintenance of cerebral microcirculation during RCP due to lack of modality of real-time measurement of cerebral microcirculation. Experimental study for monitoring cerebral circulation though retinal vessel fluorescein angiography during RCP was reported by Dong et al. [8]. They emphasized that the percentage of blood flow effectively delivered by retrograde cerebral perfusion depends on the vessel’s anatomy. We considered that as one of the reasons of the difference between the blood flow in the ONH of two patients in the present study. And we also believe that physiologic factors, including venous compliance, venous volume, valve insufficiency, affected the blood flow of RCP. The differences of these factors are difficult to detect. We believe that LSFG is meaningful because it can see the result directly, meaning relatively evaluate the blood flow. Ono et al. attempted retinal vessel fluorescein angiography during RCP in human case, and confirmed the blood flow [9]. Endo et al. observed the retinal vessel with fundus camera [10]. They calculated the ratio of the retinal vessels to the optic disc, and compared quantitatively to verify that RCP under HCA increased the ratio of the retinal vessels than only HCA. Our report is only a pilot study; however, we believe that a number of new knowledge related to cerebral microcirculation during aortic surgery will be obtained with this method.

## Conclusion

The potential of the new neuro-monitoring method, LSFG, was demonstrated in this pilot study. The LSFG could detect the blood flow in the ONH during both retrograde and antegrade cerebral perfusion. And the value was correlated with NIRS.
